# Application of Pupillometry in Neurocritical Patients

**DOI:** 10.3390/jpm13071100

**Published:** 2023-07-05

**Authors:** Chiu-Hao Hsu, Lu-Ting Kuo

**Affiliations:** 1 Division of Neurosurgery, Department of Surgery, National Taiwan University Hospital Hsin-Chu Branch, Biomedical Park Hospital, Hsin-Chu County 302, Taiwan; 2 Institute of Clinical Medicine, College of Medicine, National Taiwan University, Taipei 100, Taiwan; 3 Division of Neurosurgery, Department of Surgery, National Taiwan University Hospital, Taipei 100, Taiwan; 4 Division of Neurosurgery, Department of Surgery, National Taiwan University Hospital Yunlin Branch, Yunlin 640, Taiwan

**Keywords:** neurocritical care, outcome, pupillometry, traumatic brain injury

## Abstract

Pupillary light reflex (PLR) assessment is a crucial examination for evaluating brainstem function, particularly in patients with acute brain injury and neurosurgical conditions. The PLR is controlled by neural pathways modulated by both the sympathetic and parasympathetic nervous systems. Altered PLR is a strong predictor of adverse outcomes after traumatic and ischemic brain injuries. However, the assessment of PLR needs to take many factors into account since it can be modulated by various medications, alcohol consumption, and neurodegenerative diseases. The development of devices capable of measuring pupil size and assessing PLR quantitatively has revolutionized the non-invasive neurological examination. Automated pupillometry, which is more accurate and precise, is widely used in diverse clinical situations. This review presents our current understanding of the anatomical and physiological basis of the PLR and the application of automated pupillometry in managing neurocritical patients. We also discuss new technologies that are being developed, such as smartphone-based pupillometry devices, which are particularly beneficial in low-resource settings.

## 1. Introduction

Examination of brainstem reflexes is crucial for a comprehensive evaluation of neurosurgical patients, particularly in emergent situations. These reflexes include the PLR, corneal reflex, oculocephalic reflex, and gag reflex. The pupillary light reflex (PLR), which causes the pupil to constrict in response to bright light, is a routine assessment in the field of neurology [1] and emergency medicine [2]. Pupil size is regulated by two muscles: the circumferential sphincter muscle, which constricts the pupil in response to light and is innervated by the parasympathetic nervous system, and the iris dilator muscle, which dilates the pupil in low light intensity and is controlled by the sympathetic nervous system [3]. The integrity of the reflex arc passing through the brainstem is essential for PLR, making it a valuable tool for evaluating brainstem function. The PLR is particularly useful for monitoring cerebral dysfunction in patients with traumatic brain injuries and after neurosurgery, and deterioration of the PLR is a strong predictor of adverse outcomes after acquired brain injury [4]. 

The standard pupil examination involves assessing pupil size, shape, symmetry, and the PLR. The PLR is a strong predictor of outcome and survival after brain injury, such as traumatic brain injury (TBI) [5] or subarachnoid hemorrhage [6]. Furthermore, the shape of the pupil has been found to be significant in several intracranial pathological conditions, and adverse neurological outcomes have been associated with an oval or football-shaped pupil [7]. However, manual pupil assessment results are inconsistent and prone to inaccuracies due to variations in light intensity and exposure times with different light sources, as well as the skill levels and visual acuity of different examiners. The visual descriptions of manual PLR assessments are often subjective and imprecise, involving terms such as reactive, non-reactive, dilated, brisk, or sluggish [8]. Similar issues arise when evaluating pupil size and shape. To address these problems, modern automated pupillometry has been developed, which provides more accurate, reliable, and reproducible measurements by standardizing the distance between the light source and the eye, and the intensity of the light stimulus (Table 1).

Numerous studies have demonstrated the clinical applications of automated pupillometry in various fields, including neurosurgery, psychology, psychiatry, emergency medicine, cognitive science, ophthalmology, sleep medicine, anesthesiology, and pharmacology [9]. A thorough neurological examination is essential for providing high-quality medical care and good communication among different specialties in the treatment of neurological or neurosurgical patients. Essential neurological examinations include the evaluation of the level of consciousness, cognitive function, cranial nerve function, motor function, sensory function, and reflexes [10]. A careful and detailed neurological examination allows medical staff to localize possible lesions based on functional neuroanatomy, make differential diagnoses, determine pathogenesis, and identify the best treatment strategies. Additionally, performing serial neurological examinations, including pupillometry and the Glasgow Coma Scale (GCS), throughout the course of treatment can offer crucial insights to physicians in terms of evaluating the effectiveness of the ongoing treatment and identifying any potential pathologies at the earliest possible stage [11]. The aim of this review is to introduce the anatomy and physiology of the pupil, discuss the clinical application of automated pupillometry, and explore the prospects of automated pupillometry in neurocritical care. 

## 2. The Modulation of PLR

The pupil size is regulated by two groups of smooth muscle: the dilator iridis muscle and the sphincter pupillae muscle. The dilator muscle is innervated by the sympathetic system while the sphincter is innervated by the parasympathetic system [12]. The pathway of PLR on either side has an afferent limb and two efferent limbs. The afferent limb runs within the optic nerve (CN II), while the efferent limbs run within the oculomotor nerve (CN III). Photosensitive retinal ganglion cells, activated by light, transmit signals through the optic nerve (CN II) and across both optic tracts via the optic chiasm. From there, the signals travel to the Edinger–Westphal nuclei, which give rise to preganglionic parasympathetic fibers that innervate the sphincter pupillae muscles, causing pupil constriction.

Light stimulation in one eye causes a response in both eyes. The response of the pupil of the eye stimulated by light is called the direct light reflex, while the response of the pupil of the other eye to the light stimulation of the opposite eye is called the consensual light reflex. This reflex occurs due to the crossing over of the nasal retinal fibers at the optic chiasm, allowing each pretectal nucleus to receive signals from both eyes. The projections of each pretectal nucleus to both Edinger-Westphal nuclei contribute to the consensual light reflex.

The sympathetic system controls pupillary dilation. First-order neurons descend from the hypothalamus and synapse in the spinal cord at the T1 and T2 level. Second-order neurons then exit the spinal cord and synapse in the superior cervical ganglion. Third-order neurons then ascend with the carotid plexus and innervate the dilator iridis muscle. Horner syndrome, characterized by the triad of ptosis, miosis, and hemifacial anhidrosis, is caused by lesions affecting the first- and second-order neurons [13]. Hemifacial anhidrosis will not occur if a lesion affects only third-order neurons. 

In addition to light stimulus, changes in pupil size can also be affected by involuntary factors, such as interest, pain, sexual stimulation [14,15], uncertainty [16], decision conflict [17], mental errors, and cognitive load (which includes attention, information processing, working memory, and decision making) [18,19]. However, these changes are rarely larger than 0.5 mm [20]. In cases of brain herniation caused by mass lesion, abnormal PLR can be detected earlier than other brainstem reflexes, indicating compression of the brainstem. Pupil abnormalities usually indicate an initial stage of ongoing compression of the brainstem and have been reported to be a strong predictor of outcome and survival [21,22,23,24]. Therefore, a correct assessment of the PLR is crucial in neurocritical patients. 

Moreover, accurate measurement of PLR parameters requires attention to several factors beyond light stimulation. Medications can affect pupil size and the response to light, leading to errors in PLR measurement. Alcohol consumption has been found to affect various pupil parameters, including diameter, constriction amplitudes, and velocities, making it a reliable tool for detecting alcohol intoxication [25,26,27,28,29]. In neurodegenerative diseases, such as Parkinson’s disease [30] and Alzheimer’s disease, a significant reduction in maximum velocity of constriction (MCV) and maximum constriction amplitude (MCA) [31,32] was shown. Acetylcholine and L-DOPA are considered to be the main mechanisms underlying this effect [33,34,35]. Diabetes can also affect the PLR, with slower constriction velocity and reduced reflex amplitudes [36,37]. Age is another factor that influences pupil parameters as older individuals have reduced constriction amplitudes and velocities [38,39,40]. 

## 3. Automated Pupillometry

### Measuring PLR and Pupillary Light Dilation (PLD)

The PLR and pupillary response to an alerting stimulus are measured as changes in pupil size. Several variables of these reflexes are assessed, including latency of onset, maximum amplitude, duration, and constriction and dilation velocities. The PLR has three key components: latency of the reflex, CV, and dilation velocity (DV). The conventional method of evaluating pupil reactivity involves using a penlight or flashlight to measure pupil size and the PLR, but this method is subjective and dependent on the skills of the technician. Values of pupil response parameters may vary due to differences in light intensity and duration, making it difficult to compare measurements between different examiners or even the same examiner at different times. 

A pupillometry device comprises a digital camera, a microcomputer, an illumination source, and a monitor. In this device, infrared light with a wavelength of 850 nm is emitted towards the pupil, and a digital camera with an infrared sensitive array detects the reflected light from the iris [23,41]. Pupillometry devices provide rapid and repetitive measurements, providing data in just 3 to 4 s. These devices can yield valuable information, such as the baseline and maximum constriction size of the pupil, latency of the reflex, CV, and DV. Some algorithms, such as the Neurological Pupil index (NPi, developed by NeurOptics, Inc., Irvine, CA, USA), can compare the pupil parameters of a patient with a normative model of pupil reaction to light and automatically grade the patient’s pupillary response on an objective scale [42]. The NPi takes into account various variables, such as latency, CV, mean CV, baseline pupil size, percentage of change, and DV. A lower NPi score (less than 3) has been associated with increased intracranial pressure [43,44] as well as poorer outcomes in critically ill patients [45,46,47,48,49]. Thus, the NPi provides an objective and automated assessment of pupil parameters, thereby overcoming the subjectivity and technician-dependence associated with traditional methods of pupil assessment. 

Automated pupillometry has been shown to significantly improve the accuracy and precision of pupil measurements [50]. However, there are some potential limitations that must be taken into account. For example, a case has been reported where a patient’s pupil constricted only 1 mm with continuous light stimulation of 7–9 s, and it was later discovered that the patient was using an opioid [51]. Therefore, while automated pupillometry can provide objective and reliable measurements, traditional penlight examinations should also be used in clinical practice to strengthen accuracy. Additionally, some clinical conditions may limit the use of pupillometry, such as when a patient is unable to follow instructions due to agitation or involuntary movement. 

## 4. Clinical Application of Pupillometry

Pupillometry has found its most well-established application in the postacute care of cardiac arrest patients. The 2020 American Heart Association (AHA) Guidelines for Cardiopulmonary Resuscitation and Emergency Cardiovascular Care have been recently included automated pupillometry and the NPi as a standard and reproductible assessment when performed in conjunction with other prognostic tests [52]. Pupillometry is classified as a class IIb recommendation and can be performed at 72 h or longer after cardiac arrest to aid in predicting the neurological outcome in patients who remain comatose. 

## 5. Role of Pupillometry in Neurocritical Care

Pupillometry has emerged as a crucial tool in neurocritical care, enabling clinicians to detect elevated intracranial pressure and impending neurological deterioration at an early stage [44,46,53,54,55,56,57]. Pupil evaluation using a penlight for reactivity and a pupil gauge for size is often performed, but the results are often heterogenous and inconsistent, with variations in the intensity and duration of ambient light, as well as with different examiners. Furthermore, subjective and imprecision description of the PLR, including terms such as reactive, non-reactive, dilated, brisk, or sluggish contribute to the lack of consistency in repeated measurements. A previously observational study by Chen and Colleagues [42] revealed only moderate inter-rater reliability between practitioners for pupil size, shape, and reactivity. 

In comparison to manual pupil assessment, pupillometry offers a more accurate and objective assessment, with only one-third of non-reactive pupils [24,58,59,60,61] and half of anisocoria cases [8] being detected by practitioners. Additionally, concordance between the pupillometer and subjective assessment of pupil parameters was poor, with an 18% discordance in PLR and 39% discordance in measurements with small pupils (diameter < 2 mm) [8].

## 6. Prognostic Indicator in Intracranial Pathology

Several studies have demonstrated the potential use of pupillometry as a prognostic indicator, particularly in cases of cardiac arrest and acute brain injuries. Taylor et al. [62] prospectively analyzed collected pupillometry data from 117 patients with various acute brain injuries, including aneurysmal subarachnoid hemorrhage and spontaneous intracerebral hemorrhage, and found that the NPi score on admission was significantly different between patients with poor and favorable progress (Glasgow Outcome Scale (GOS) 0.88 ± 1.68 vs. 3.89 ± 0.97, *p* < 0.001). An initial NPi cutoff of 3.4 was able to predict outcome at 1 month with a specificity of 84.6% and sensitivity of 86% [62]. The observational study by Lee et al. [63] in their hospital-onset unresponsive patients found that brain herniation syndrome could be detected with a NPi cut-off set below 1.6 with a specificity of 91% and a sensitivity of 49%. The study also found that the NPi was negatively associated with in-hospital mortality (odds ratio (OR): 0.77; 95% CI: 0.62–0.96) and poor neurological outcomes (modified Ranking scale ≥ 4) at 3 months (OR: 0.67; 95% CI: 0.49–0.90) after adjustments. Another three case series by Papangelou et al. showed that the NPi can be used to predict transtentorial brain herniation, with 73% of NPi values being abnormal (NPi < 3) prior to the occurrence of transtentorial herniations [64]. These studies suggest the potential value of pupillometry as a prognostic tool in neurocritical care. 

The ORANGE (Outcome Prognostication of Acute Brain Injury using the Neurological Pupil Index) study is an ongoing, prospective, observational, and international cohort study that began in 2020 [65]. The study aims to enroll at least 420 patients with acute brain injuries, including TBI, aneurysmal subarachnoid hemorrhage (aSAH), and intracerebral hemorrhage (ICH). The primary objective of the study is to investigate the relationship between the NPi and 6-month mortality or as well as poor neurological outcome, defined as a score of 1–4 on the Extended GOS. The ORANGE study is expected to provide valuable information on the prognostic value of pupillometry in acute brain injury patients, which could ultimately lead to better management and treatment decisions for these patients. 

## 7. Increased Intracranial Pressure

Intracranial pressure (ICP) monitoring is an important method for evaluating and managing patients with neurocritical conditions. While intracranial catheter placement remains the gold standard for measuring ICP, several non-invasive techniques are being advocated to provide useful information, including optic nerve sheath diameter, pulsatility index, estimated ICP using transcranial Doppler, and the NPI [66]. Pupillometry, which assesses changes in pupil size and reactivity, has been increasingly recognized as an effective tool for detecting ICP. Several studies have been conducted in the past on the association between pupillometry and increased ICP. 

The relationship between increased ICP and pupillometry (decreased NPi) was first described in a case series back in 2002 [67], and subsequent studies have confirmed this association [68,69,70]. In 2003, Taylor et al. conducted a study involving 310 normal volunteers and 26 patients with acute brain lesions [23]. They found that a CV falling below 0.6 mm/second might be a useful cut point for detecting increased ICP. Additionally, they noted that when patients had diffuse brain swelling without a midline shift, the CV started to drop when ICP increased above 30mmHg. An asymmetry of the pupil size greater than 0.5 mm was also recognized when the ICP increased above 20 mmHg [23]. 

Another observational cohort study with 54 patients of severe TBI demonstrated that during sustained and elevated ICP (>20 mmHg for more than 10 min), the NPi would decrease concomitantly (baseline 4.2 ± 0.5 vs. 2.8 ± 1.6, *p* < 0.0001). An abnormal NPi can also imply an unfavorable 6-month outcome (15% in GOS 1–3 vs. 0% in GOS, 4–5 patients; *p* = 0.002). Furthermore, the study showed that an increase in the baseline NPi was associated with a reduction in ICP after osmotherapy was administered.

In 2018, Ong et al. published a prospective observational cohort study of 72 patients with a total of 402 pupillometry measurements [71]. They studied the effects of osmotic therapy (20% mannitol or 23.4% hypertonic saline) on pupil reactivity and found that improved pupil reactivity was noted within 2 h after osmotic therapy administration. Etiologies of increased ICP included intraparenchymal hemorrhage (36.1%), traumatic brain injury (15.3%), anterior circulation stroke (13.9%), brain tumor (11.1%), posterior circulation stroke (9.7%), and subarachnoid hemorrhage (6.9%). The effect could last for an average of 5 h and was most notable in patients with abnormal NPi before treatment (*p* = 0.0235). The relationship between ICP and pupillometry has been well-established in the literature. 

## 8. Traumatic Brain Injury

Quantitative pupillometry has been increasingly recognized as a valuable non-invasive tool for detecting ICP in patients with acute brain injuries. The measurement of the PLR has been used to triage patients with TBIs and evaluate the severity of the injury. 

El Ahmadieh et al. found that an NPi < 3 in comatose patients with a GCS score ≤ 8 could indicate that the PLR pathway is compromised, and the patient might require intervention even when the brain CT did not show signs of herniation or midline shift [72]. Singer et al. sought to develop a non-invasive method to evaluate the severity of TBIs and measure ICP [73]. They found that optic nerve sheath diameter and pupillometry were suitable supplementary screening tool for severe TBIs. The study demonstrated that patients with severe TBIs had a significantly large optic nerve sheath diameter and decreased NPi compared to controls. In addition, the study also found that the patients with low NPi values had a higher likelihood of requiring intervention, such as craniotomy or ICP monitoring. 

## 9. Intracerebral Hemorrhage (ICH)

Recent studies have evaluated the use of pupillometry in predicting increased ICP and the correlation between computed tomography (CT) indicators of intracerebral hemorrhage and pupillometry parameters. Giedde-Jeppe et al. [57] conducted a retrospective study on 23 sedated nontraumatic supratentorial ICH patients. The study found that PLR parameters, such as CV, DV, latency, and percentage change of aperture, had high negative predictive values (around 97% to 99.2%) but low positive predictive values (only 7.2% to 8.3%) for predicting increased ICP (defined as >20 mmHg). These findings suggest that automated pupillometry may facilitate the avoidance of routine invasive ICP monitoring. In addition to the clinical outcomes and ICP of hemorrhagic stroke, CT indicators of ICH were also reported to be correlated with the NPi. The ICH volume exhibited the most significant correlation with the NPi, especially the ipsilateral pupil (r^2^ = 0.48, *p* < 0.0001). Moreover, the horizontal midline shift of the septum pellucidum (r^2^ = 0.25, *p* = 0.0006) and shift of the pineal gland (r^2^ = 0.21, *p* = 0.0017)] on the CT image were also correlated with the NPi. Thus, the use of automated pupillometry may facilitate the avoidance of routine invasive ICP monitoring. Additionally, CT indicators of ICH, such as ICH volume and midline shift, have been shown to be correlated with pupillometry parameters. 

## 10. Aneurysmal Subarachnoid Hemorrhage

Aneurysmal subarachnoid hemorrhage is a serious condition that requires careful monitoring of neurological function. Several studies have been conducted on the use of pupillometry in evaluating disease severity and predicting clinical outcomes in patients with aSAH. Natzeder et al. collected 4456 NPi data points from 18 patients with aSAH and found that the mean NPi tended to be lower in patients with clinically severe (World Federation of Neurological Surgeons (WFNS) grade 4–5) compared with non-severe (WFNS grade 1–3) aSAH (3.75 ± 0.40 vs. 4.56 ± 0.06, *p* = 0.171) [74]. The mean NPi also tended to be lower in patients with an unfavorable outcome (GOS 1–3) compared to those with a favorable outcome (GOS 4–5) at the time of discharge (3.64 ± 0.48 vs. 4.50 ± 0.08, *p* = 0.198). However, the application of the mean NPi to predict severity and outcome was not statistically significant. The study did find a statistically significant difference in the frequency of pathological NPi values in clinically severe (WFNS grade 4–5) and non-severe (WFNS grade 1–3) aSAH (16.3% ± 8.8% vs. 0.0% ± 0.0%, *p* = 0.002). Pathological NPi values were also noted more frequently in patients with unfavorable outcomes compared to those with favorable outcomes (19.2% ± 10.6% vs. 0.7% ± 0.6%, *p* = 0.017) [75]. In summary, the study demonstrated that the frequency of pathological NPi values was significantly different between severe and non-severe aSAH patients and between patients with favorable and unfavorable outcomes.

Ortega-Perez et al. studied the data of 82 aSAH patients with a total of 4403 pupillary readings [6]. The authors found that correlation between standard deviation of PLR values and discharge modified Rankin scale (MRS) was moderate and negative (r = −0.3 to −0.47, *p* < 0.01), which means that a higher variation in pupillometry readings was associated with better outcomes at discharge.

Delayed cerebral ischemia (DCI) is a potential cause of neurological deficits occurring several days after aSAH. Early detection of DCI and vasospasm can help clinicians to intervene earlier and achieve better neurological outcomes. Aoun et al. conducted a study on 56 aSAH patients with 635 paired pupillometry and transcranial Doppler (TCD) measurements [74]. They found a significant association between DCI and sonographic vasospasm (*x*^2^ = 6.4112, *p* = 0.0113, OR = 1.6419), as well as between DCI and abnormal decrease in the NPi (*x*^2^ = 38.4456, *p* < 0.001, OR = 3.3930). The odds ratio for the NPi to predict DCI was higher than that for sonographic vasospasm (3.39 vs. 1.64). Out of the 12 patients who had DCI, 7 patients showed a decrease in NPi to an abnormal range, and in 71% of patients, this decrease could be detected more than 8 h before neurological deterioration. The NPi normalized in all patients after treatment of vasospasm. Although this study was limited by the small sample size, it provided early evidence on the potential utility of pupillometry in predicting outcomes and detecting DCI and vasospasm.

## 11. Non-Convulsive Status Epilepticus

Research about pupillometry in seizure is relatively limited compared to other neurocritical diseases. Compared with other types of seizure, making the correct diagnosis of non-convulsive status epilepticus (NCSE) in a neurocritical unit is much more challenging. Godau et al. [76] found a reduction in the NPi or a significant difference between the left and right NPi might be a useful quantitative modality to evaluate NCSE. Another study also conducted by Godau revealed the potential of the NPi to assess treatment responses of NCSE [45]. In addition to the NPi, decreased dilation velocity (DV) was also discovered to have association with unreactive electroencephalography (EEG) signals, which is a possible utility in patients with seizure or critical illness [77].

## 12. Ischemic Stroke

The Establishing Normative Data for Pupillometer Assessment in Neuroscience Intensive Care (END-PANIC) registry [78] is a database of pupillometry data that has been used to evaluate the relationship between pupillometry and various neurocritical conditions. The authors measured the midline shift of septum pellucidum in serial brain images and collected pupillometric data from 134 patients with acute ischemic stroke and intracerebral hemorrhage (70.1% ischemic, 29.9% hemorrhagic) from the END-PANIC registry. They found a significant correlation between the midline shift, the NPi (left (*p* < 0.001), right (*p* < 0.001)), coefficient of variation (left (*p* < 0.005), right (*p* < 0.001)), and pupillary asymmetry (absolute difference between right and left; *p* < 0.05). However, there was no significant correlation between the midline shift and pupil size.

Peinkhofer et al. enrolled 74 patients with acute ischemic stroke and assessed them 24 h after endovascular recanalization therapy [79]. Interestingly, the correlation between pupillary size and CV was found in controls and left hemispheric infarction, but the correlation was absent in right hemispheric infarction. 

Malignant cerebral edema is a devastating complication following ischemic infarction of the large vessel territory. One retrospective study [80] found that ipsilateral NPi abnormality was associated with malignant cerebral edema (OR = 21.80, *p* = 0.007). Another study by Cortes et al. also revealed that abnormal NPi values were associated with acute cerebral edema [81]. In addition, a lower NPi value (3.88 ± 0.65 vs. 4.45 ± 0.46, *p* < 0.001) and a sudden decrease in the NPi (29.5% vs. 11.1%, *p* = 0.006) were significant predictors of neurological worsening during follow-up, with all patients with NPi values below 2.8 developing neurological deterioration [75]. 

Delirium is another common complication following ischemic stroke, and dysregulation of the autonomic nervous system is thought to be the culprit. One recent cohort study [82] with 64 patients of acute ischemic stroke found that the DV of PLR during delirium was lower than that without delirium (unadjusted difference was −0.22 mm/second; 95% CI = −0.42 to −0.01, *p* = 0.041). The literature supports the use of pupillometry in predicting malignant cerebral edema, neurological worsening, and delirium.

## 13. Pain Assessment in Neurocritical Care

A self-rated score is the most common and intuitive method to assess patients’ pain intensity. However, in certain circumstances, such as facing noncommunicating patients, and patients who are usually critically ill, consciousness-disturbed, or mechanically ventilated, a self-rated score is impractical. A noxious stimulus could evoke sympathetic system and lead to pupillary dilation. As a result, pupillometry had been applied to investigate as an indicator of pain and effect of analgesia [9,83,84]. Specifically, Guglielminotti et al. [85] reported the utility of pupillometry in assessment of the degree of analgesia by epidural anesthetics for pregnant patients suffered from labor pain and uterine contraction. In addition to adult patients, pupillometry was also reported as a useful and effective tool for assessment of pain in the sedation of pediatric patients [86].

## 14. Smartphone-Based Pupillometry

Pupillometry has been established as a reliable method for examining patients’ pupils in various clinical settings. However, its cost and accessibility can pose obstacles, particularly in low-resource or developing countries. To address this, several studies have focused on developing smartphone-based pupillometry, which offers a promising alternative approach that is more affordable and accessible (Table 1). 

A pilot study in 2013 introduced a prototype system of smartphone-based pupillometry, using an optical apparatus with infrared (λ_max_ = 850 nm) and white LEDs (λ_max_ = 570 nm) attached to a smartphone’s camera [87]. Infrared LEDs were used to take the mydriasis image in the dark without affecting pupil size, while the white LED was used to cause miosis. All the images were processed with Photoshop (Adobe Systems Inc., San Jose, CA, USA) through a proposed algorithm in the Android system. The results showed that the accurate rate was high (97.7 ± 1.3%), and smartphone-based pupillometry was worth researching and developing. 

Piaggio et al. also published a study confirming the higher accuracy and significant correlations for all the pupillometry-related measures compared to a commercial solution [88]. Another system named Sensitometer test (KagenAir LLC, Appleton, WI, USA) with an infrared camera system based on the iPhone (Apple Inc., Cupertino, CA, USA) was introduced by McAnany et al., which showed a good level of agreement between the use of a smartphone and that of a dedicated pupillometer for pupil assessment (r = 0.91, *p* < 0.001) [89]. These studies suggest that smartphone-based pupillometry could provide a reliable, simple portable, and cost-efficient method for pupil measurements. This technology could have particular value in low-resource settings. However, further research and development are needed to fully realize the potential of this approach. 

## 15. Limitations

PLR is one of the most important examinations in neurocritical care. Automated pupillometry provides an accurate, reliable, and reproducible measurements, which overcome the drawbacks of manual PLR assessment. Numerous studies have proved the practicability in different fields. In this review, we focused on the application in neurocritical care and organized the updated literatures. 

Although there was increasing evidence aiming at the utility of pupillometry, most of it was from small-group studies. Another limitation is that baseline pupil size and reflex are inconsistent in different populations. Furthermore, the device, light source, light intensity and duration, and disease status were also discrepant. Moreover, different studies used different variables, including the NPi, DV, CV, and latency. As a result, these factors make data interpretation and comparisons across studies difficult. Automated pupillometry has the potential in clinical practice, but we should apply it with caution.

## 16. Conclusions

Pupillometry has become an important tool in the management of intracranial pathology, with increasing evidence supporting the role of automated pupillometry. Although there are currently no guidelines regarding the routine application of pupillometry, automated pupillometry offers several advantages over traditional subjective manual pupil examination. It provides more precise, objective, and quantitative measurements, with better reliability, and reproducibility. Furthermore, automated pupillometry can detect subtle and early changes in pupil size more accurately, which can aid in early diagnosis and intervention. Further research is needed to establish standardized guidelines for the use of pupillometry in clinical practice and to explore its potential for improving patient outcomes in a variety of intracranial pathologies.

## Figures and Tables

**Table 1:** Comparison of automated pupillometers.

Product	NPi^®^-300 Pupillometer	NPi^®^-200 Pupillometer	NeuroLight^®^	2WIN (with Dynamic Pupillometry APP)	PupilScreen System
Manufacturer	NeurOptic (Irvine, CA, USA)	NeurOptic (Irvine, CA, USA)	IDMED (Marseille, France)	Adaptica (Padova, Italy)	University of Washington
Design	Hand-held	Hand-held	Hand-held	Hand-held	Smartphone app and a container box
Light source	LED	LED	LED	LED	Smartphone flashlight
Camera	Infrared	Infrared	Infrared	Infrared	Smartphone camera
Pupillary assessment scale	Neurological Pupil index™ (NPi^®^)		Quantitative Pupillary Light Reflex (QPi)	nil	nil
	0: Non-reactive, immeasurable, or atypical response <3.0: Abnormal/“Sluggish” 3.0–4.9: Normal/“Brisk”		0–2: Non-reactive/week 3: Reduced 4–5: Good/very good		
Detection time	2–3 s	3 s	4 s	14 s	Not Announced
Size accuracy	0.03 mm	0.03 mm	0.1 mm	0.1 mm	0.2 mm
Pupil-size threshold	0.08–10 mm	1.0–10 mm	1.0–9.9 mm	4–11 mm	Not Announced
Latency time limit	0–0.5 s	0–0.5 s	0.1–0.4 s	Not Announced	Not Announced
Weight	344 g	320 g	280 g	840 g	Depends on smartphone
Estimate price		~300 USD	~500 USD	~5000 USD	NA
Other features	Wireless charge				

## Data Availability

Data is contained within the article.
